# Mitochondrial Calcification

**DOI:** 10.20900/immunometab20210008

**Published:** 2021-01-29

**Authors:** Bhargavi Duvvuri, Christian Lood

**Affiliations:** Department of Medicine, Division of Rheumatology, University of Washington, Seattle, WA 98195, USA

**Keywords:** mitochondria, calcification, calcium, phosphate

## Abstract

One of the most fascinating aspects of mitochondria is their remarkable ability to accumulate and store large amounts of calcium in the presence of phosphate leading to mitochondrial calcification. In this paper, we briefly address the mechanisms that regulate mitochondrial calcium homeostasis followed by the extensive review on the formation and characterization of intramitochondrial calcium phosphate granules leading to mitochondrial calcification and its relevance to physiological and pathological calcifications of body tissues.

## BACKGROUND

Calcification or mineralization, an accumulation of insoluble calcium salts in tissue, is a key biological process that is physiologically restricted to hard tissues, including bone, teeth, and the hypertrophic zone of growth plate cartilage [1]. This essential biological process becomes pathological when the calcium salt deposition occurs in soft tissues including skin, muscles, arteries, and lungs [2–5]. Major determinants for calcification to occur either extracellularly or intracellularly are the concentrations of free calcium ions (Ca^2+^) and inorganic phosphate (Pi), the presence of suitable membrane where mineralization can be initiated and the relative amounts of factors that promote or inhibit calcification. Extracellularly such a high calcium and phosphate ionic environment is observed in the matrix vesicles (MV) released from cells, including osteoblasts and chondrocytes [6]. MVs contain mineralization-promoting cargo, including specific lipid profiles of vesicular membrane that promote Ca^2+^ entry into MV and its binding with high affinity and enzymes like tissue non-specific alkaline phosphatase increasing the ionic concentrations of Pi in the MV lumen by hydrolyzing phosphate substrates [7]. Intracellular calcification is mainly mediated by mitochondria [8], which play a crucial role in maintaining cellular calcium homeostasis by scavenging excessive cytosolic Ca^2+^ as Ca-P complexes. In both contexts, key two phases of mineralization include the accumulation of calcium and phosphate ions to promote nucleation and crystal formation usually of hydroxyapatite (HA) [Ca_10_(PO_4_)_6_(OH)_2_] in nature followed by the exposure of these preformed apatite material to extracellular fluid promoting the crystal proliferation and thus beginning the process of mineralization and crystal deposition.

## MITOCHONDRIAL CALCIUM TRANSPORT

**Figure 1.**
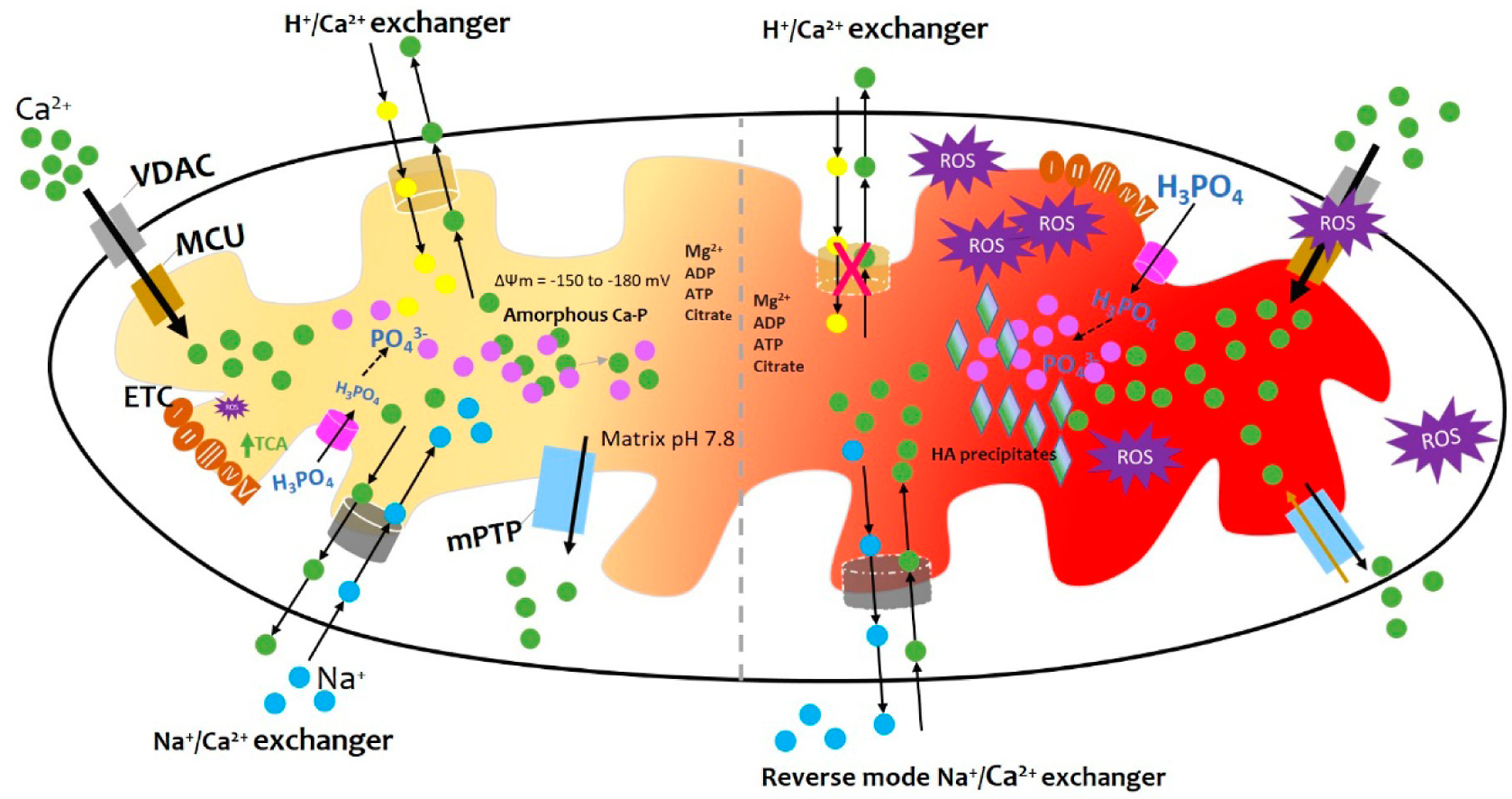
Summary of mitochondrial calcification. As detailed in the main text, physiological levels of mitochondrial Ca^2+^ result from highly regulated Ca^2+^ influx and efflux mechanisms, including buffering by the formation of calcium phosphate complexes. The formation of amorphous calcium phosphate complexes is promoted by the alkaline pH of the mitochondrial matrix and undefined nucleation factors. The crystallization of calcium phosphate into hydroxyapatite is prevented by factors such as magnesium ions, ATP, ADP, citrate, and polyphosphates. However, in the conditions of inflammation, hypoxia, and injury an imbalance of calcium influx and efflux ensues filling mitochondria with amorphous calcium phosphate complexes and crystalline hydroxyapatite granules. Details in the text. *Figure concept adapted from* [12].

Since the focus of this viewpoint is mitochondrial calcification rather than calcium regulation of mitochondria, in this section we will briefly address important aspects of mitochondrial calcium uptake and efflux including mechanisms that regulate mitochondrial calcium homeostasis (Figure 1). For more information on this broad scientific area readers are referred to some exhaustive reviews [9–11].

## MITOCHONDRIAL CALCIUM UNIPORTER (MCU)-DEPENDENT MITOCHONDRIAL Ca2^+^ UPTAKE

The direct evidence that mitochondria rapidly accumulate Ca^2+^ is known since the 1960s [10–13]. The movement of Ca^2+^ ions in and out of mitochondria is a concerted activity of ion transporters on outer mitochondrial (OMM) and inner mitochondrial membranes (IMM). The OMM is highly permeable to various ions, including Ca^2+^, whose transport is mediated by a non-selective porin, voltage-dependent anion channel [14,15]. In contrast, calcium entry through IMM into the matrix is facilitated primarily by a highly calcium selective channel, mitochondrial calcium uniporter (MCU), located in the IMM [16–18]. MCU exhibits lower affinity for Ca^2+^ (Kd around 10–20 μM) and higher conductance rates than Ca^2+^ uptake channels in the endoplasmic reticulum (ER), which makes them suitable to respond to large increases in cytosolic Ca^2+^ that occur physiologically at the calcium release or the entry points of ER and plasma membrane calcium channels and to the pathological Ca^2+^ overload [19]. The MCU-mediated Ca^2+^ entry into mitochondria is an electrogenic process driven by the steep mitochondrial membrane potential, Δψm, (~150–180 mV, the mitochondrial matrix is negative) across the IMM established by the respiratory chain or by the reverse mode ATP synthase activity [13]. Accordingly, proton ionophores such as *p*-(trifluoromethoxyl)-phenyl-hydrazone (FCCP) that dissipate Δψm suppresses mitochondrial Ca^2+^accumulation. Whereas selective inhibitors of MCU, mainly ruthenium red-based compounds, and small-molecule inhibitor DS16570511 directly inhibit Ca^2+^ uptake [20–22]. Despite the enormous thermodynamic pull, Ca^2+^ levels in mitochondria are maintained at the resting levels (~100 nM), suggesting the presence of mechanisms that maintain the baseline levels of mitochondrial Ca^2+^ by directly regulating the activity of MCU [23]. These regulating mechanisms are critical to ensure that MCU acts as a gate-keeper to prevent channel opening at resting cytosolic Ca^2+^ levels, thus avoiding deleterious/futile calcium cycling and matrix overload and allowing prompt response of mitochondrial calcium uptake in situations of cytosolic Ca^2+^ increase. MCU, a 40 kDa protein, functions as a tetramer where a single protomer is composed of two transmembrane domains (TM1 and TM2) joined by a highly conserved short loop facing an intermembrane face (IMS) and N- and C- domains facing mitochondrial matrix. The motif between TM1 and TM2 characterized by negatively-charged residues (DIME motifs) serves as a selectivity filter of the MCU channel [24,25]. MCU does not have classical Ca^2+^-sensing domains, hence cannot regulate its own activity. Indeed, the activity of MCU is regulated by EF-hand-containing Ca^2+^ binding proteins, mitochondrial calcium uptake 1 (MICU1) and mitochondrial calcium uptake 2 (MICU2) found in the IMS, along with IMM protein essential MCU regulator (EMRE). A study suggested that by exerting opposing effects, MICU1 and MICU2 heterodimers fine-tune the activity of MCU, where at lower cytosolic Ca^2+^ levels the dominant inhibitory effect of MICU2 shuts down the MCU activity; however, conformational change induced in dimers by increases in cytosolic Ca^2+^ releases MICU2-dependent inhibition of MCU triggering MICU1-mediated augmentation of MCU channeling activity [26]. EMRE [27], a transmembrane protein is critical for the assembly of functional MCU, and promotes MCU interaction with regulatory subunits, MICU1 and MICU2 and thus contributes to channel gating. Also, MCU, a paralog of MCU, was demonstrated to be an endogenous dominant-negative subunit of MCU that greatly impairs Ca^2+^ ion permeation properties of MCU [28]. Interestingly, the expression and relative proportions of MCUb vary significantly among tissues contributing to the tissue-specific variations of the mitochondrial calcium uptake rates observed in different mammalian tissues. For example, skeletal muscle exhibits high MCU: MCUb ratio, which matches with its high mitochondrial calcium conductance rates [28,29], compared to adult heart that exhibit relatively elevated expression of MCUb resulting in considerably low MCU activity [28,29]. In cardiac cells, with 37% of cell volume being mitochondria, such regulation through the higher expression of MCUb is crucial to prevent the massive accumulation of Ca^2+^ by mitochondria and dysfunction and undesired cytosolic Ca^2+^ buffering preventing heart contractile activity. Further, induction of MCUb expression was shown to be a stress-responsive mechanism to overcome calcium overload following cardiac injury [30]. Future studies in this area should explore how various physiological and pathological stimuli alter the ratios of MCU: MCUb and the consequences of such an altered expression on mitochondrial Ca^2+^ uptake sensitivities/loading capacity of tissues and implications for tissue calcification. Two additional MCU regulators are MCU regulator 1 (MCUR1) and solute carrier family 25 member 3 (SLC25A23). Silencing of MCUR1 abrogated MCU-dependent mitochondrial Ca^2+^ uptake in both basal and stimulated conditions [31] and was critical for full assembling MCU via its interaction with MCU and EMRE [32]. SLC25A23, an IMM protein with ATP-Mg/Pi carrier function, represents another regulator of MCU given that silencing of SLC25A23 reduced MCU activity and thus Ca^2+^ influx into mitochondria following stimulation [33]. Although MCU is considered to be the predominant mechanism of mitochondrial Ca^2+^ uptake, interestingly MCU-KO mice had significantly reduced but detectable levels of matrix Ca^2+^ with only relatively minor alterations in the functions dependent on mitochondrial influx of Ca^2+^: mitochondrial respiration and basal metabolism. The only substantial defect is a decrease in skeletal muscle peak performance, indicating that in vivo alterations in matrix Ca^2+^ are most important for adapting to needs of higher energy demands as in strenuous muscle work [34,35]. Overall, the observations from MCU-KO mice suggest the presence of additional MCU-independent Ca^2+^ uptake mechanisms in mitochondria [36–41]. In addition, the possibility that in the absence of MCU, mitochondrial Ca^2+^ efflux mechanisms work in reverse mode, thus bringing Ca^2+^ into the matrix rather than exporting Ca^2+^, cannot be ruled out [34]. For an extensive summary on genetic manipulations of MCU and effects on mitochondrial Ca^2+^ uptake and phenotypes in different cell lines and species, see reference De Stefani et al. [42].

## MCU-INDEPENDENT MITOCHONDRIAL Ca2^+^ UPTAKE

Other potential Ca^2+^ uptake pathways reported in mitochondria include the rapid mode Ca^2+^ uptake (RaM), [41,42], and Ca^2+^ influx through mitochondrial ryanodine receptor 1 (mRyR1) functioning in excitable cells [43,44] among others [14,40]. Of these multiple mitochondrial Ca^2+^ influx mechanisms, RaM, described as a kinetic model of Ca^2+^ uptake, operates very rapidly (hundred times faster than MCU; [37]) and responds to transient and low cytosolic Ca^2+^ pulses of <200 nM. Its conductivity is brief, which is inhibited at extramitochondrial Ca^2+^ levels greater than 200 nM by Ca^2+^ binding to an external inhibition binding site before undergoing resetting by drop in external Ca^2+^ levels [38]. Fast uptake of Ca^2+^ can nevertheless create transient sites of high matrix Ca^2+^ that can activate ADP phosphorylation [15,16]. However, the levels are not sufficient enough for global cytosolic Ca^2+^ buffering and the induction of mitochondrial permeability transition pore (mPTP), a large pore in the inner mitochondrial membrane that increases the mitochondrial permeability to solutes up to 1.5 kDa whose persistent opening can lead to cell death [17]. Hence, the evolution of RaM seems to be in the regulation of the rate of oxidative phosphorylation by generating brief, high free matrix Ca^2+^ levels with relatively small amounts of Ca^2+^ [37]. Such a mode of transient, rapid, and low mitochondrial Ca^2+^ uptake may be more relevant to tissues like a heart with very short but frequent Ca^2+^ pulses, thus protecting them against matrix Ca^2+^ overload the opening of mPTP but still activating Ca^2+^-sensitive metabolic reactions. mRyR1, mainly characterized in excitable cells like cardiac muscle cells, is another fast Ca^2+^ uptake pathway in mitochondria that is active in the micromolar ranges (10–50 μM) of Ca^2+^ and is inactivated at higher concentrations [39]. Since mRyR1, unlike MCU, has relatively low selectivity for Ca^2+^ with high conductance rates, it can rapidly dissipate Δψm. This energetically unfavorable process is prevented presumably with a lower number of mRyR1 on single mitochondria, so that membrane depolarization is localized and is quickly corrected by metabolic activity [14,40]. The unique Ca^2+^ dependence of various Ca^2+^ influx channels suggests their specific roles in different cytosolic Ca^2+^ environments of different tissues. However, modulation of their function in (patho) physiological conditions remains to be explored.

For the mitochondrial calcification, Ca^2+^ uptake via MCU seems to be a major mechanism, since RaM and mRYR1, although they have high conductivity [37], are operational transiently around physiological or at modest elevations of extramitochondrial Ca^2+^ [38] unlike MCU that operates even in the conditions of more extended and higher cytosolic Ca^2+^ pulses with relatively slow conductance, thus mediating large amounts of matrix Ca^2+^ accumulation necessary for calcification.

## MITOCHONDRIAL Ca2^+^ EFFLUX PATHWAYS

### Na^+^ Dependent Mitochondrial Ca2^+^ Efflux

As to the efflux mechanisms, Ca^2+^ can be exported from the matrix via Na^+^-dependent or independent mechanisms. The Na/Li/Ca exchanger (NCLX) in the inner mitochondrial membrane [18,43,44], ubiquitously found in most cell types and particularly robust in excitable cells, catalyzes the exchange of Na^+^ or Li for Ca^2+^. Although the precise stoichiometry for NCLX still unclear, the general consensus has been an influx of 3 Na^+^ per 1 Ca^2+^ efflux, indicating that NCLX is also electrogenic [45] similar to its counterparts (Na^+^/Ca^2+^ exchanger, NCX) in the plasma membrane. The unique feature only shared with mitochondrial NCLX being Li^+^-mediated Ca^2+^ transport in addition of Na^+^/Ca^2+^ exchange [46], hence the name NCLX instead of NCX. Since Ca^2+^ influx into mitochondrial matrix is driven by negative electrochemical gradient, the influx process is energetically downhill, and the efflux is uphill that would require energy. The minimum energy required for the export of 1 mole of Ca^2+^ from mitochondria is calculated to be 33.04 kJ/mol [47]. Energy requirements for such a transport could be met by ATP hydrolysis, energy from ETC activity via oxidation of substrates, or coupling the Ca^2+^ efflux to another ion that is moving down its electrochemical gradient or some combination of these energies. Na^+^ ion, whose matrix concentrations are maintained lower than cytosolic Na^+^ levels by a Na^+^/H^+^ exchanger [19], meets such an energy requirement. Hence, the large negative Δψm coupled with Na^+^ gradient i.e., lower inside, provides the driving force for extruding Ca^2+^ from matrix against its gradient through NCLX. The electrogenic feature of NCLX would indicate that in depolarized mitochondria, NCLX would function in reverse mode mediating the influx of Ca^2+^ rather than extrusion [23]. Despite the profound effect of NCLX on mitochondrial Ca^2+^, as shown in gene silencing and overexpression experiments [44], NCLX does not affect the steady-state resting level mitochondria Ca^2+^. This would suggest the low affinity of NCLX to Ca^2+^ [48] and its prominent role during rapid and robust matrix Ca^2+^ changes to restore mitochondrial Ca^2+^ levels to baseline.

### Na^+^ Independent Mitochondrial Ca^2+^ Efflux

In non-excitable cells, Ca^2+^ efflux is primarily mediated by H^+^/Ca^2+^ exchanger [49]. In a genome-wide Drosophila RNA interference screen, leucine zipper EF-hand-containing transmembrane protein 1 (LETM1), previously described as K^+^/H^+^ exchanger, was identified as a molecule that fulfills the criteria of mitochondrial Ca^2+^/H^+^ exchanger with an electrogenic stoichiometry of 1H^+^/Ca^2+^ [50]. However, this electrogenic stoichiometry for LETM1 is different from unequivocally established electroneutral 2H^+^/Ca^2+^ for this antiport [51]. Interestingly, LETM1 mediates Ca^2+^ influx in an electrogenic manner (one Ca^2+^ in for one H^+^ out) when matrix Ca^2+^ is low, but when mitochondrial Ca^2+^ is high or cytoplasmic pH is low, LETM1 mediates Ca^2+^ efflux [50].

While the exact kinetics of these mitochondrial efflux mechanisms may vary significantly between tissues, overall, the kinetics of efflux rate is always much slower than influx, and this kinetic imbalance is apparent from Vmax values of these mechanisms. For example, initial studies established Vmax of MCU to be 1400 nmol Ca^2+^ (mg protein)^−1^ min^−1^ compared to combined Vmax of 20 nmol Ca^2+^ (mg protein)^−1^ min^−1^ for efflux mechanisms [52]. This kinetic imbalance leads to two questions: (1) Why is the efflux rate through these Ca^2+^ selective mechanisms slower than influx? (2) How do mitochondria overcome pathological matrix Ca^2+^ overload that could ensue since the influx rate exceeds that of combined Ca^2+^ selective efflux pathways? Ca^2+^ accumulation by mitochondria is a function of extramitochondrial Ca^2+^ levels [53]. Hence a higher efflux rate would mean higher cycling of Ca^2+^ across the IMM, which will be met at the expense of increased proton conductance manifesting as a decrease in proton electrochemical gradient and hence in increased respiration, suggesting that respiratory capacity would be spent on Ca^2+^ recycling [54]. Thus, having low *V*max and easily saturable efflux pathways would limit the energy to be spent on mitochondrial Ca^2+^ cycling. However, such a kinetic imbalance would expose mitochondria to a threat of Ca^2+^ overload, which can be overcome by the opening of a high conductance channel in the IMM such as mPTP that shows a prominent dependence on matrix Ca^2+^ for its activation [17,55]. The mPTP open and closed transition states are modulated by various endogenous effectors, and the consequences of pore opening vary dramatically based on the open time [56]. mPTP is a large, non-selective channel, which in its fully open state has a permeability cutoff for molecules up to 1500 Da. Thus, with a long term opening, transport of ions and molecules occurs between mitochondria and cytosol followed by the influx of water resulting in mitochondrial swelling. Eventually, OMM ruptures with the release of proapoptotic proteins from mitochondrial IMS into the cytosol, potentially leading to apoptotic cell death or necrosis.

Interestingly, transient openings or “flickerings” of mPTP have been reported, suggesting that mPTP may also play a physiological role in Ca^2+^ efflux. Thus, mPTP is also considered to be one of the important matrix Ca^2+^ efflux mechanisms [55]. Unlike other mitochondrial Ca^2+^ efflux mechanisms, mPTP is not selective for Ca^2+^. Such an ion non-selectivity may facilitate a unique advantage to mPTP in overcoming the opposition by diffusion potential (−30 mV) that is generated across the IMM due to Ca^2+^ efflux through Ca^2+^ selective channels. Thus, in the absence of compensating ion transport, i.e., the influx of positive charges and efflux of negative charges, the efflux of Ca^2+^ through Ca^2+^ selective channels would be extremely slow. One way to overcome the magnitude of diffusion potential and subsequently to increase the rate of Ca^2+^ efflux is to increase the IMM permeability, for example, by increasing the H^+^ conductance. The ion non-selectivity of mPTP allows the charge compensation within a single channel itself at zero potential, thus allowing the rapid efflux of Ca^2+^ from matrix regulated by the modulation of the mPTP open time. Since there is no concentration gradient for Na^+^ and K^+^ across IMM, mPTP is, in a way, selective for Ca^2+^ transport from mitochondria [56]. Given the low affinity of mPTP Ca^2+^ binding sites (Kd 25 μM), the Ca^2+^ concentration required for the activation of mPTP is relatively higher than the concentration for ADP phosphorylation (20 nmol/mg vs 4 nmol/mg protein), suggesting that higher matrix Ca^2+^ overload is required for pore activation [57]. Matrix modulators like elevated levels of mitochondrial reactive oxygen species (mtROS) can decrease the amount of Ca^2+^ required for mPTP activation in pathological conditions. It should be noted that pore opening itself can also contribute to the generation of mtROS [58]. Other mPTP inducing agents include Pi, oxaloacetate, and acetoacetate, while adenine nucleotides and Mg^2+^ are common endogenous inhibitors of mPTP activation, including acidic pH and high membrane potential [56]. Incidentally, membrane depolarization and increase in matrix pH subsequent to Ca^2+^ overloading promote the activation of mPTP.

The depolarization of Δψm in turn, results in the reversal of mitochondrial F0-F1 ATP synthase, thus promoting ATP hydrolysis. Since Mg^2+^ has a ten-fold higher ATP affinity, ATP hydrolysis would increase the matrix Mg^2+^ levels [59]. The combination of these events would increase the concentrations of mPTP activation inhibitors (Mg^2+^ and ADP), leading to pore closure restoring Δψm. This would explain the basis for transient openings of mPTP in vivo (as detailed in the review, Bernardi [56]. Another possibility for mPTP flickering could be during rapid Ca^2+^ influx through RaM and mRyR1, where mPTP at these high Ca^2+^ microdomains could be activated, leading to Ca^2+^-induced Ca^2+^ release (discussed in Gunter and Sheu [60]). Depending on the matrix Ca^2+^ load, released Ca^2+^ can trigger Ca^2+^ uptake into adjacent mitochondria.

## MITOCHONDRIAL Ca2^+^ UPTAKE AND CROSSTALK WITH ROS

The major targets of mitochondrial Ca^2+^ are rate-limiting enzymes of tricarboxylic acid (TCA) cycle that are activated in different mechanisms: isocitrate dehydrogenase and ketoglutarate dehydrogenase are directly activated by Ca^2+^ binding whereas pyruvate dehydrogenase (PDH) activation depends on Ca^2+^-regulated PDH phosphatase [10,61]. The activation of TCA boosts the synthesis of reducing equivalents, NADH and FADH2, substrates of electron transport chain (ETC), thus enhancing the ETC activity and subsequent increase in proton-gradient. In addition, mitochondrial Ca^2+^ also stimulates the activities of adenine nucleotide transporter [62] and complex V (mitochondrial F_0_F_1_ ATP synthase) [63], which by harnessing proton gradient generates ATP. Overall, a rise in matrix Ca^2+^ in response to an increase in cytosolic Ca^2+^, which invariably is associated with stimulated cells, allows mitochondria to decode the energy demands of cell stimulation and adjust ATP synthesis accordingly. Since mitochondrial ETC is one of the main sites that generate cellular reactive oxygen species (ROS) in physiological and pathological conditions, Ca^2+^ accumulation in the matrix during cellular activation can directly contribute to mtROS by promoting mitochondrial metabolism. Mitochondrial Ca^2+^ also activates nitric oxide synthase, whose product nitric oxide inhibits complex IV enhancing mtROS generation [56]. Matrix Ca^2+^ overload in conjunction with oxidative stress activates the opening of mPTP. The opening of mPTP results in the rapid collapse of Δψm and membrane depolarization resulting in increased mtROS. An independent study has shown that Ca^2+^ induces ROS via Ca^2+^-mediated complex II disintegration by binding to cardiolipin, a principle IMM anionic lipid that promotes complex II stability. However, when bound by Ca^2+^ in the conditions of matrix overload, cardiolipin coalesces into separate homotypic clusters releasing the enzymatically competent sub-component of complex II that generates ROS by transferring electrons from succinate to molecular oxygen [64]. Oxidative stress, in turn, stimulates mitochondrial Ca^2+^ overload by mPTP. Available evidence shows that various calcium transport systems are sensitive to redox conditions [65]. This includes oxidants that impair Ca^2+^ influx into endoplasmic reticulum and extrusion from the plasma membrane via inhibition of sarco (endo) plasmic reticulum Ca^2+^-ATPase [66,67] and plasma membrane Ca^2+^-ATPase, respectively [68,69] complemented by increased release from endoplasmic reticulum Ca^2+^ stores [70,71]. The resultant increase in the cytosolic Ca^2+^ causes transient opening of mPTP to prevent cell from cytosolic overload but stimulating mitochondrial Ca^2+^ overload. Interestingly, in in vitro conditions, inflammation and hypoxia-induced oxidative stress were shown to regulate MCU-mediated mitochondrial Ca^2+^ uptake independent of cytosolic Ca^2+^ by relieving it from gatekeeping of MICU1/ MICU2, thus resulting in augmented mitochondrial Ca^2+^ at baseline cytosolic Ca^2+^ [57]. Specifically, in the conditions of enhanced mtROS, conserved cysteine residue in the NTD of MCU undergoes redox modification (*S*-glutathionylation) that induces a conformational change MCU promoting high order oligomerization and persistent activation even in resting conditions despite the presence of functional MICU1/MICU2 [57]. The increased MCU activity with a constitutive elevation of mitochondrial Ca^2+^, in turn, led to overproduction of mtROS, perturbed mitochondrial bioenergetics, and apoptosis. Overall, these data suggest that Ca^2+^ and ROS create a self-perpetuating cascade that can culminate in the mitochondrial Ca^2+^ overload and perturbed cell functions [59]. Further, in the conditions of oxidative stress, Na^+^/Ca^2+^ exchangers, the Ca^2+^ efflux mechanisms function in a reverse mode promoting calcium influx rather than efflux of matrix Ca^2+^ [72].

## MITOCHONDRIAL MATRIX CALCIUM BUFFERING: FORMATION OF CA-P COMPLEXES

Mitochondrial matrix Ca^2+^ modulates various processes, including stimulation of aerobic mitochondrial metabolism, suppression of autophagy, regulation of cell life/death processes and Ca^2+^-induced Ca^2+^ feedback, cytosolic Ca^2+^ buffering, and in regulating spatial restriction of Ca^2+^ waves (discussed in the review, Patron et al. [26]. Thus, the maintenance of matrix Ca^2+^ levels is essential, which is a function of Ca^2+^ influx and efflux across the mitochondrial membranes, including the buffering of Ca^2+^. Mitochondrial Ca^2+^ buffering capacity expressed as the ratio of total and free Ca^2+^ is in the range of 30,000 to 150,000 respectively, for physiological and pathological conditions, suggesting the enormous importance of organelle’s Ca^2+^ buffering [73–75]. Net uptake of Ca^2+^ into mitochondria is coupled to the co-transport of Pi, resulting in the formation of Ca-P complexes [18–21]. Since mitochondria, unlike endoplasmic reticulum [76] do not have specialized Ca^2+^ binding proteins, complex formation with Pi is considered a major mechanism of buffering matrix Ca^2+^ contributing to mitochondria’s massive calcium storage ability [22–24]. In fact, it was shown that there exists a linear relationship between total and free calcium levels below 10 nmol Ca^2+^/mg of mitochondrial protein, but beyond which (in the range of 1–5 μM) the matrix-free calcium remains invariant due to buffering by calcium phosphate [75]. Consistently, depletion of mitochondrial Pi resulted in the loss of mitochondrial calcium homeostasis with uncontrolled matrix-free Ca^2+^ levels [75]. Pi enters into matrix through phosphate carrier (PiC) or phosphate transporter whose main physiological role is to function as a Pi:H^+^ symport. The PiC transports the Pi, which is equivalent to its fully protonated form H_3_PO_4_ with H^+^. Since the phosphate form that interacts with matrix Ca^2+^ is PO4^3−^, the phosphate has to undergo three stepwise deprotonations (H_3_PO_4_ to H_2_PO_4_^−^ to HPO_4_^2^^−^ to PO_4_^3−^) and thus the concentration of Pi in matrix is inversely proportional to the third power of proton gradient in the matrix. As we know that Ca^2+^ accumulation in matrix decreases Δψm which is compensated by the net expulsion of H^+^ by respiratory chain. If this were the only way, Ca^2+^ accumulation will eventually stop since entire Δψm will be converted to proton gradient (ΔpH). Note that Ca^2+^ influx into mitochondria is driven by Δψm component of proton motive force. However, Pi’s transport with increasing ΔpH (provided the presence of external Pi) will neutralize the increasing matrix pH facilitating the Ca^2+^ accumulation and the formation of reversible Ca-P complexes by transported Pi with accumulated matrix Ca^2+^ [77]. At around ten nmol Ca^2+^ mg protein^−1^ in the mitochondrial matrix, there is a kinetic balance between influx and efflux where the efflux pathway becomes independent of matrix Ca^2+^ called set point since it is at this concentration Ca-P complexes begin to form thus buffering matrix Ca^2+^ [75]. It should be noted that these Ca-P complexes are osmotically inactive, thus preventing mitochondrial matrix swelling as ion accumulation progresses [26].

## NATURE OF MITOCHONDRIAL CALCIUM SALT COMPLEXES

As demonstrated in both isolated and in situ brain mitochondria, robust Ca^2+^ accumulation induced by extramitochondrial Ca^2+^ levels beyond the set point causes the formation of electron-dense granules within the matrix [78,79]. These electron-dense intra-mitochondrial Ca-P granules are amorphous and have both organic and inorganic constituents. Based on the method of granule isolation, the organic moiety accounts for about 16–60% of Ca-P granule content represented by nitrogen, protein, and sugar ribose, suggesting the presence of RNA [80]. Chemical analysis revealed that Ca^2+^ and Pi are the major inorganic constituents of matrix Ca-P granules primarily corresponding to hydroxyapatite and whitelockite or a mixture of both as shown by the X-ray diffraction patterns of microincinerated granules (inducing the crystallization). Also, significant traces of MgO presumably derived from MgCO3 were also found [80]. Similar to precipitates analyzed in the context of biomineralization [81], the composition of mitochondrial precipitates seems to be complex both in structure and composition. Based on the Ca/P ratios ranging from 1.0 to 1.67, stoichiometric compounds of Ca and P reported in mitochondria include various forms of calcium orthophosphates [80,82,83] as shown in Table 1. Many of Ca-P complexes identified in the mitochondrial matrix are known to spontaneously interconvert based on the Ca/Pi ratios and energy availability [83,84]. The rate of mitochondrial Ca^2+^ accumulation seems to be one of the majorfactors affecting the stoichiometry of calcium phosphate complexes, where faster Ca^2+^ infusion rates promote higher Ca/P ratios (~1.5, Ca_3_ (PO_4_)_2_) as shown in rat liver mitochondria [83]. Findings from electron microscopy and X-ray analysis of Ca^2+^-loaded mitochondria and the fact that Ca-P precipitates of crystalline nature are not observed in live cells reveal the indefinite amorphous nature of Ca-P complexes suggesting that crystallization is held in check within the mitochondrial matrix [30]. The amorphous nature of dense mitochondrial granules containing Ca-P was also confirmed with samples prepared by cryo-scanning transmission electron tomography, which overcomes the limitations associated with dehydrated or heavy-metal staining samples [85]. Further, the dissociation of Ca-P complexes upon mitochondrial depolarization and their release from respective transporters confirms the reversible nature of these granules [86]. Thus, allowing the gradual exit of calcium and Pi from mitochondria through their respective carriers once the cytoplasmic calcium storm subsides [22,34]. The indefinite amorphous nature of matrix Ca-P was attributed to endogenous mineralization inhibitors such as citrates and magnesium ions, ATP and ADP within the mitochondria matrix [31–33]. In addition to these endogenous inhibitors, polyphosphates (polyP, (P_n_O_3n+1_)^(n+2)−^) expressed by mitochondria can also inhibit the formation of insoluble Ca-P complexes or precipitates, thus regulating the levels of free Ca^2+^ in the mitochondrial matrix. PolyP are negatively charged polyanions formed by the polymerization of many Pi molecules [87], which are known as potent inhibitors of Ca-P precipitation in vitro [88]. Accordingly, cells overexpressing mitochondrially targeting polyP hydrolyzing enzyme called polyphophatase (MitoPPX cells) have decreased levels of free matrix Ca^2+^ despite similar loading of Ca^2+^ uptake compared to wild type (independent of Ca^2+^ efflux rates), suggesting the buffering of matrix Ca^2+^ as Ca-P insoluble clusters [89,90]. This conclusion is supported by microscopic data where an increased accumulation of electron-dense granules was seen in MitoPPX cells compared to wild type cells under both basal and stimulated conditions [90].

## INTRAMITOCHONDRIAL AGGREGATES

In a detailed study of experimentally induced mitochondrial calcification, both apatite-like crystalline, needle-shaped aggregates, and granular aggregates have been identified [91–93]. Consistent withelectron-dense granules of Ca^2+^ overloaded mitochondria, intramitochondrial aggregates had both inorganic and organic components (glycoproteins, lipids). Interestingly, the type of intramitochondrial inorganic aggregates differed based on the tissue type examined in the study. Crystalline aggregates were restricted to apparently normal muscular and myocardial cells, and granular aggregates were mainly found in swollen mitochondria of degenerated hepatic cells. However, the relationship between the state of cells and the type of intramitochondrial aggregates is less evident in literature, which requires examination at the early stages of mitochondrial calcification. In general, consistent with the presence of crystallization inhibitors, crystalline aggregates are less commonly found in mitochondria, and they have been reported in both normal and damaged cells. Granular aggregates are mostly widely reported both in the context of mitochondria overloaded with Ca^2+^ and in mitochondria of normal cells and cells at various stages of degeneration. In this study, although, morphologically both aggregate forms seem to be very closely associated with mitochondrial cristae there were some differences during the early stages of calcification. Crystalline aggregates were more closely situated near cristae membranes, unlike granular aggregates, which are close to cristae but lie more in the matrix. This association of crystalline aggregates with membranes is interesting considering the affinity of anionic phospholipids to Ca^2+^ and their potential role as organic components aiding in the deposition of inorganic material and in the initiation of mineralization [94]. Further, no relationship was found between these two aggregate forms as only rarely granular and crystalline structures were found in the same aggregate, and mitochondria with one or two crystalline structures were found without any apparent granular aggregates [92]. Mitochondria filled with granular structures representing supersaturated ratios of Ca/Pi did not show any crystalline structures. Although results from this study suggest that intramitochondrial crystalline aggregates can form directly in the absence of granular intermediates, the process of mitochondrial calcification may be similar to bone calcification involving phases of nucleation and crystal growth, respectively [95–97]. According to classic nucleation theory, the major energy barrier for crystal growth is the formation of the critical nucleus (nucleation stage), which will support the growth and proliferation of crystals by adding more ions or nuclei clusters. Nucleation to occur de novo in the solution will require the respective ion concentrations to exceed their solubility properties (i.e., critical supersaturation). However, pre-nucleation clusters or surfaces that resemble crystal nucleus facilitate nucleation even at biological concentrations, thus overcoming the energy barrier of nucleation. For mitochondria, such quasi-stable pre-nucleation structures promoting the formation of apatite-like structures could be amorphous tricalcium phosphate or maybe even brushite [95,96,98,99]. Once this intermediate, obligatory step of forming insoluble Ca-P precipitates is achieved, HA crystals can form involving the poorly characterized complex process of crystal growth by adding more ions in the context of ongoing matrix Ca^2+^ and Pi overload.

## EFFECT OF CALCIFICATION ON MITOCHONDRIAL FUNCTION

Since both crystalline and granular aggregates are closely associated with mitochondrial cristae they could affect mitochondrial function, namely mitochondrial metabolism and ROS production. Matrix free Ca^2+^ overload induces mtROS generation. However, very little is known about how the formation of Ca-P precipitates affects mitochondrial function. Ca-P granules effecting mitochondrial respiration were demonstrated in a study where the activity of complex I was inhibited, thus decreasing the rate of ATP synthesis. It was proposed that Ca-P precipitates could be forming physical barriers isolating complex I from its substrate, NADH [100]. However, it remains to be explored why complex I, but not other respiratory complexes, are inhibited by such Ca-P precipitation.

## CELLULAR REACTION TO CALCIFIED MITOCHONDRIA

In an experimentally induced calcification of rat myocardium where focal areas of calcification were restricted to mitochondria, severely calcified cells generated cellular reaction [91]. In that study, cells of macrophagic type surrounded calcified areas and were seen to be engaged in active phagocytosis. Such a prompt inflammatory reaction seems to be important in preventing calcification from spreading to surrounding structures since only myocardial cells but not interstitial, and collagen fibers were involved in the calcification [91]. Neutrophils could be another potential phagocytic cell type involved in the cellular reaction to calcified cells. In an inflamed muscle tissue of patients with JDM, we have demonstrated infiltrating neutrophils and macrophages adjacent to calcified tissue involving in the engulfment of seemingly indigestible calcium crystals potentially of mitochondrial origin [101,102]. Since calcified mitochondria can potentially be harmful to cellular health, such calcified mitochondria could be extruded out of the cell as a protective mechanism to prevent cellular damage. However, if phagocytes do not promptly clear extruded calcified mitochondria, it could also result in ectopic calcification under a pro-calcifying environment and additionally could also induce a pathological crystal-mediated inflammation [101,102].

## MITOCHONDRIAL CALCIFICATION IN HEALTH AND DISEASE

The role of mitochondrial granules in biological mineralization has been reported [103–108]. Incidentally, the early discovery of how cells load calcium into the matrix vesicles leading to chondrocyte growth plate calcification is based on the findings that significant amounts of accumulated mitochondrial calcium get transferred to MVs in the form of mitochondrial granules [8,14,15]. Similar proposition has been made for bone mineralization based on the temporal relationship between mitochondrial granule depletion and the mineralization front, suggesting that calcium and phosphate ions for bone mineralization are stored in mitochondrial granules [104,108]. But evidence directly linking intramitochondrial granules with vesicles participating in extracellular mineralization process has been missing. More recently, a direct evidence on the role of mitochondrial granules in extracellular mineralization has been demonstrated where calcium-containing vesicles were identified conjoining with calcium phosphate containing mitochondria, suggesting Ca-P granule storage and transport processes [106]. According to the proposed model, mitochondrial Ca-P granules are first transferred to intracellular vesicles possible by diffusion, which is not unusual for mitochondria given the evidence of vesicular transport between mitochondria and other cellular organelles [109,110]. These intracellular vesicles loaded with amorphous calcium phosphate are then transported to extracellular space propagating into apatite-like structures in extracellular matrix initiating mineralization [106].

Unlike metastatic calcification, which is caused by the increased substrate availability, dystrophic calcification is secondary to the altered membrane integrity due to trauma or inflammation and as such is observed at the sites of tissue degeneration. Mitochondria could be the initial sites for intracellular calcification in both types of calcification considering their robust Ca^2+^ uptake and storage abilities. In case of metastatic calcification, elevated levels of extracellular calcium and phosphate ions could lead to increasing levels of these ions within the cell. Although some of these ions will be exported out of the cell via efflux mechanisms on the plasma membrane, but over the time ions will accumulate in mitochondria forming Ca-P complexes, thus initiating the process of intracellular metastatic calcification. In case of dystrophic calcification, despite the presence of normal levels of calcium and phosphate ions in circulation, increased plasma membrane permeability due to injury, inflammation or hypoxia make expulsion of ions from the cell ineffective leading to their accumulation in mitochondria initiating the process of intramitochondrial mineral formation. Mechanistically, mitochondrial calcification can be the contributing factor for soft-tissue calcification of dystrophic type as observed in many pathological conditions including dermatomyositis, scleroderma, systemic lupus erythematosus, and mixed connective tissue diseases, some of in which mitochondria have been implicated [5,60–65]. However, there is still lack of definitive evidence of mitochondrial calcification in these disease conditions and its role in disease. The observation that inflammation and the associated mitochondrial oxidative stress leading to pathological mitochondrial Ca^2+^ overload even in baseline cytosolic Ca^2+^ levels has important implications for diseases like juvenile dermatomyositis in which dystrophic calcifications of muscle and skin are associated with chronic inflammation [111]. Incidentally, there is emerging evidence that mitochondrial calcification in skeletal muscle cells subsequent to inflammation is driven by excessive mtROS [101], warranting further studies on how various pathophysiological stimuli can cause dysregulated mitochondrial Ca^2+^ uptake and calcification.

To summarize, mitochondrial calcification is a physiological process to protect cells from calcium-induced cytotoxicity; however, dysregulated may contribute to disease and calcification of tissues. Hence, understanding mechanisms regulating mitochondrial calcification and its role in accumulation of extracellular calcium deposits in tissue may allow for identification of novel therapeutic targets in several diseases, including dermatomyositis [5,102,112].

## Figures and Tables

**Table 1.** List of calcium orthophosphate compounds characterized in mitochondria [82,83].

Compounds	Form of calcium orthophosphate	Chemical formula	Ca/P ratio
**Stoichiometric**	Dicalcium phosphate dihydrate	CaHPO_4_∙H_2_O (Brushite)	1.0
	Octacalcium phosphate (OCP)	Ca_3_(HPO_4_)_2_(PO_4_)_4_∙5H_2_O	1.33
	α-tricakium phosphate (α-TCP)	Ca_3_(PO_4_)_2_ (whitelockite)	1.5
	Hydroxyapatite (HA)	Ca_10_(PO_4_)_6_(OH)_2_	1.67
**Non-Stoichiometric**	Amorphous calcium phosphate		~1.45

## ABBREVIATIONS

ADP: adenosine diphosphate
ATP: adenosine triphosphate
EMRE: essential MCU regulator
FCCP: *p*-(trifluoromethoxyl)-phenyl-hydrazone
HA: hydroxyapatite
IMM: inner mitochondrial membrane
IMS: intermembrane face
LETM1: leucine zipper EF-hand-containing transmembrane protein 1
MCU: mitochondrial calcium uniporter
MCUR1: MCU regulator 1
MICU1: mitochondrial calcium uptake 1
MICU2: mitochondrial calcium uptake 2
MitoPPX: mitochondrially targeting polyP hydrolyzing enzyme
mPTP: mitochondrial permeability transition pore
mRyR1: mitochondrial ryanodine receptor 1
mtROS: mitochondrial reactive oxygen species
MV: matrix vesicles
NADH: micotinamide adenine dinucleotide hydrogen
NCLX: Na/Li/Ca exchanger
OMM: outer mitochondrial membrane
PDH: pyruvate dehydrogenase
PiC: rhosphate carrier
RaM: rapid mode Ca^2+^ uptake
SLC25A23: Solute carrier family 25 member 3
